# Antiseptic and Antipiretic Treatment of Phthisis

**Published:** 1888-04

**Authors:** 


### ANTISEPTIC AND ANTIPYRETIC TREATMENT OF PHTHISIS.

Dr. W. H. Spencer, in a paper read before the Bath and Bristol Branch of the British Medical Association, gives the complete records of two cases of acute tubercular phthisis, treated antiseptically and antipyretically, one of which died of profuse hæmoptysis just as recovery was almost assured ; the other recovered completely. Both cases passed into acute tubercular phthisis from acute pneumonia.

In the first case a mixture of sulphuric acid, bark, and ether was used pretty continuously throughout the case, and along with the other remedies. Cod-liver oil was taken at intervals and for short periods, but it was not well borne. Kairin and quinine were used as antipyretics. The former always quickly reduced the temperature, and effectually ; but, as is usual with all antipyretics of its class, the fall of temperature was transient, and the resulting depression was marked. Quinine was used in large doses at intervals only, and for a short time, without any effects as to the general progress of the case. For general treatment in the second case, a mixture of sulphuric acid and quinine (four grains), or one of perchloride of iron and quinine, was used almost throughout the case. Cod-liver oil was borne well, and taken for a considerable time. Salicylic acid and quinine were used as antipyretics. The former produced good effects as regards steady and continuous reduction of temperature. But, notwithstanding its combination with ether, the depressing effects were sometimes a source of anxiety. A special feature in the treatment of these cases was the method of using quinine, in moderate doses, to control pyrexia—a method attended with unqualified success.

The local treatment consisted in the use of, first, iodoform, which was given in pill form in doses of one grain, six grains in twenty-four hours. It was given for long periods in both cases. In the second case it was given alone, and was well tolerated. In the first case it was combined with one grain of quinine, and one-eighth grain of hydrochlorate of morphine, but no advantage was gained. On more than one occasion the attempt was made to increase the dose to two grains, but this always caused pain and gastric distress. The iodoform was taken by this patient for nine months continuously, with the exception of two weeks. Secondly, inhalation of vapor of the oil of eucalyptus. This was used only in the second case, and here along with the iodoform. The vapor was inhaled by means of a celluloid respiratory inhaler, worn continuously except when taking food or sleeping. No nausea or other unpleasant effect resulted. Finally, a liberal diet was allowed throughout the cases. As soon as the patients began to experience the good effects of the iodoform, the appetite improved remarkably, and good food was supplied without stint. From an early period after the return of the appetite some stout was taken with the dinner.

As regards the general principles of the treatment, everyone knows what they are. But as regards the special methods of the treatment by iodoform and eucalyptol, or both together, let me say :

1. I see no reason to doubt that, when iodoform is given in doses which the stomach will tolerate well, and given frequently and continuously for long periods, it is absorbed into the circulation ; and in the lungs, in whatever form it be, manifests its antiseptic (shall I say also antibacillary ?) properties. The good effects of iodoform so administered, in phthisical conditions, is too unequivocal to be gainsaid, however they may be produced.

2. I see no reason to doubt that, when the vapor of the oil of eucalyptus (or other antiseptic vapor that can be tolerated equally well) is inhaled continuously and for long periods, it reaches the residual air in the lungs, and so externally, as it were, bathes the affected tissues or suppurating cavities that may be open to the ingress of the air.

3. That so, I apprehend, we may have antiseptic remedies, not antagonistic, brought up on two sides to the sites of inflammatory lung lesions, or the sites of bacillary activity ; and these antiseptics, mutually co-operative, do affect for good both the inflammatory process and the bacillary activity, and bring about repair by the mode of organization after suppuration or fibroid substitution.

4. I think it is both correct and desirable to treat the pyrexia of acute phthisical processes, whether the temperature be high or moderate, by and for itself. I think no other special antipyretic than quinine should be used in phthisis ; and quinine serves other purposes as well when used as an antipyretic in moderate doses.—*British Medical Journal*, January 28, 1888.

